# Shaping the future EHDS: recommendations for implementation of Health Data Access Bodies in the HealthData@EU infrastructure for secondary use of electronic health data

**DOI:** 10.1093/eurpub/ckaf033

**Published:** 2025-09-10

**Authors:** Lise S Svingel, Caroline E Jensen, Gitte F Kjeldsen, Maria H Pedersen, Dipak Kalra, Christian F Christiansen, Katrine H Vad

**Affiliations:** Department of Clinical Epidemiology, Department of Clinical Medicine, Aarhus University and Aarhus University Hospital, Aarhus, Denmark; CONNECT - Center for Clinical and Genomic Data, Aarhus University Hospital, Central Denmark Region, Aarhus, Denmark; Central Denmark EU Office, Brussels, Belgium; Department of Innovation and International Affairs, Aarhus University Hospital, Aarhus, Denmark; Central Denmark EU Office, Brussels, Belgium; The European Institute for Innovation through Health Data, Gent, Belgium; Department of Clinical Epidemiology, Department of Clinical Medicine, Aarhus University and Aarhus University Hospital, Aarhus, Denmark; CONNECT - Center for Clinical and Genomic Data, Aarhus University Hospital, Central Denmark Region, Aarhus, Denmark; Danish Health Data Authority, Copenhagen, Denmark

## Abstract

European Union (EU) Member States face challenges in using health data for secondary purposes, constrained by inconsistent digital health systems and limited cross-border sharing. One aim of the European Health Data Space (EHDS) is to facilitate secondary health data use through the HealthData@EU infrastructure and Health Data Access Bodies (HDABs). This article provides recommendations essential for HDAB implementation, informed by the HealthData@EU Pilot project. From October 2022 to December 2024, Work Package 4 gathered insights from the HealthData@EU Pilot project, including from technical work packages and use cases, and complementary insights from the members of the HDABs Community of Practice and the External Advisory Board. Data collection involved workshops, interviews, and questionnaires, with thematic analysis guided by the EHDS user journey and the World Health Organization’s National eHealth Strategy Toolkit. Recommendations cover infrastructure, services, and interoperability. Each Member State should designate HDABs to manage secondary health data use and facilitate cross-border access. National infrastructure components deployed by HDABs and National Contact Points and a metadata catalogue compliant with the newly developed HealthDCAT-AP specification are advised to support data discovery, with a common data access application form to streamline the data permit application process. Harmonized validation procedures are recommended for ensuring high data quality and semantic interoperability. Implementation of HDABs within the HealthData@EU infrastructure represents an important step towards accessible health data for secondary use across the EU. Effective implementation requires collaboration at both national and EU level, addressing remaining ambiguities for HDAB functionality within the EHDS framework.

## Introduction

Many public and private organizations in Member States of the European Union (EU) currently face considerable challenges in the secondary use of health data, such as for research, innovation, policymaking, and regulatory activities, both nationally and internationally. Major obstacles are the substantial heterogeneity in national digital healthcare development across Member States and inadequate infrastructures, which hinders cross-border data sharing [1–3]. These obstacles impede access to diverse health and health-related data, limit public health surveillance, and restrict the exploration of health variations across the EU [2–5].

**Figure 1.**
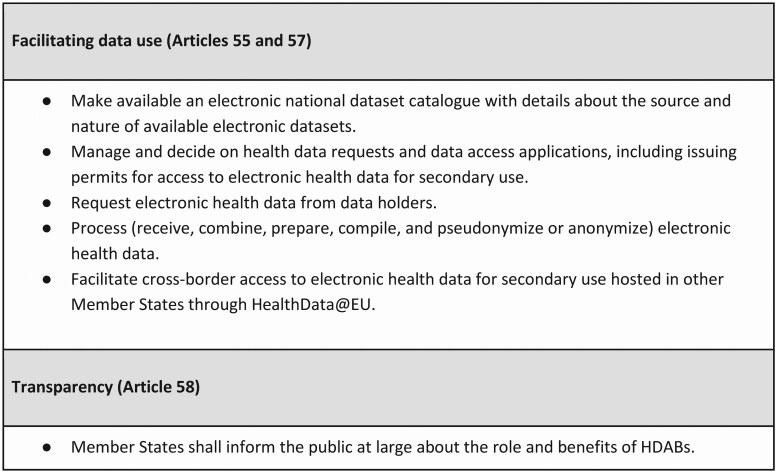
Tasks of the Health Data Access Bodies tested in the HealthData@EU Pilot.

Emerging from the broader European Strategy for Data, aiming to create a single market for data to enhance Europe’s global competitiveness and data sovereignty, the European Health Data Space (EHDS) is set to be the first of the Common European Data Spaces [6, 7]. A key objective of the EHDS is to facilitate cross-border access to electronic health data for both primary and secondary purposes [1, 8]. While developments of national health data ecosystems are vital for the generation of high-quality health data for both primary and secondary use, the development of the HealthData@EU infrastructure is fundamental to facilitate the secondary use of such health data across borders [8]. A central requirement of the EHDS Regulation is the creation of new national entities known as Health Data Access Bodies (HDABs) [1]. These HDABs will serve as intermediaries between health data holders and users, responsible for facilitating the secondary use of health data, monitoring compliance, ensuring transparency, promoting cooperation, and reporting on activities [1, 8]. Several of these tasks (outlined in Fig. 1) have been tested in the HealthData@EU Pilot project (the Pilot) that developed initial components of the HealthData@EU infrastructure [9].

To ensure Member States with varying levels of digital healthcare maturity can successfully implement the EHDS Regulation [1], there is a need for clear, actionable recommendations relating to enabling factors for the key functions and services to be upheld by HDABs. These recommendations must be based both on requirements mandated by the EHDS Regulation [1] and those necessary for the optimal functioning of HDABs, while allowing flexibility for national variations. The horizontal Work Package 4 (WP4) of the Pilot was specifically tasked with gathering insights from across the project to inform these recommendations [9].

### Objective

This article aims to present recommendations relating to key enabling factors for the implementation of HDABs within the HealthData@EU infrastructure, drawing from the work conducted by WP4 of the Pilot.

## Methods

### Setting and design

The activities of WP4 of the Pilot were conducted from October 2022 to December 2024. WP4 was designed as a horizontal work package, aimed at synthesizing the project’s achievements into recommendations for the implementation of HDABs in the HealthData@EU infrastructure [9, 10].

### Participants and sources of information

To capture a range of perspectives, including those of future HDABs and health data users, the work built upon insights gathered from various target groups both within the Pilot consortium and among external stakeholders (Supplementary Fig. S1). The main insights from contributors in the Pilot consortium were acquired from leaders of four technical work packages (WPs 5–8) and from participants, leaders, and management of five use cases (WP9) [9, 11]. The consulted external stakeholders included an External Advisory Board (EAB) with members from both private and private–public organizations [12], the HDABs Community of Practice (HDABs-CoP) with members from competent authorities and affiliated entities [13], and the national authorities from three Member States (Croatia, Denmark, and Ireland), selected to represent different levels of digital healthcare development as described in the European Commission’s Impact Assessment on the European Health Data Space [3]. Moreover, the Pilot leads (WP1) and European Commission delegates from DG Santé contributed by framing the work towards the recommendations to align with both the Pilot project deliverables and the EHDS Regulation [1], being negotiated during the project period. The legal foundation for these recommendations was the EHDS Regulation, which builds upon several existing directives and policies and was adopted by the European Parliament in December 2024 [1, 14–17].

### Data collection and analysis

The consultation process was tailored to capture the perspectives of each target group and included stakeholder engagement through meetings, workshops, questionnaires, and interviews (a list of WP4 activities is included in Supplementary Table S1) [10]. The combined activities sought to consider relevant aspects with a focus on enabling factors within topics informed by the National eHealth Strategy Toolkit from the World Health Organization (WHO Toolkit) (Supplementary Fig. S1) [18].

The perspective of future HDABs, which may also be data holders, was captured in HealthData@EU Pilot project consortium meetings, and in workshops and semi-structured interviews with each of the four technical work packages of the Pilot. To ensure a comprehensive understanding of national, international, public, and private contexts, these insights were complemented by EAB consultations and engagement with members of the HDABs-CoP by workshops, questionnaires, and semi-structured interviews with three national authorities participating in the HDABs-CoP (Supplementary Table S1) [10, 13, 19]. These interviews were transcribed, preserving linguistic nuances, and analysed using a data-driven coding approach, allowing themes to emerge directly from the data. The data were then condensed into broader thematic categories using a constant comparative method. The questionnaires, distributed to all members of the HDABs-CoP, received 20 responses from 18 different national authorities. Descriptive statistics were used to identify key trends and patterns, highlighting similarities and differences in HDAB implementation. The consultation activities were supplemented by desk research to consider the work conducted in other projects and initiatives as part of the preparatory work of the EHDS implementation.

The perspective of health data users was informed by engagement with HealthData@EU Pilot use case participants, leaders, and management via meetings, workshops, and a series of questionnaires [10, 11]. Among the use case leaders and participants were researchers from public sector institutions, research infrastructures, and EU agencies. The aim of this consultation was to gather practical experiences of encountered challenges and possible solutions when attempting to access and use electronic health data for secondary purposes in multinational projects. To this end, focus was on how the HealthData@EU infrastructure, and HDAB services in particular, may best assist data users in accessing and working with electronic health data for secondary purposes.

**Figure 2.**
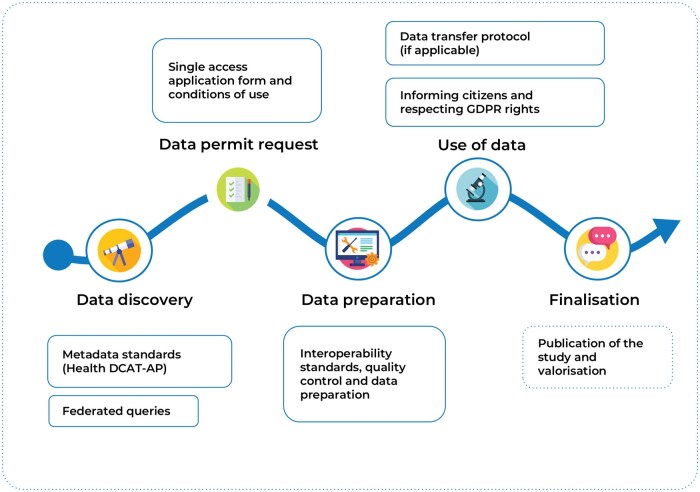
The European Health Data Space user journey (reprinted with permission from the HealthData@EU Pilot).

The EHDS user journey, a modification of the user journey identified in the joint action TEHDAS1 [20], was applied as an analytical framework to structure the insights obtained from the Pilot target groups according to the steps of secondary use of health data (Fig. 2). This framework was chosen to facilitate extraction and synthesis of learnings across target groups, as it was widely applied across work packages of HealthData@EU Pilot and largely aligns with the obligations and tasks of HDABs as laid out in the EHDS Regulation [1].

The aggregated data were interpreted with the purpose of providing actionable recommendations. For the recommendations to reflect the wide spectrum of aspects relevant to the implementation of HDABs, the recommendations were evaluated according to topics derived from the WHO Toolkit [18]. The recommendations were categorized as primary or secondary based on legal requirements, necessity based on Pilot learnings, and implementation timeline. This categorization aimed to ensure a clear understanding of the urgency and strategic importance of each recommendation to facilitate effective implementing actions. Thus, primary recommendations encompassed recommendations regarding elements that are mandatory under the EHDS Regulation and/or outcomes from the Pilot that are essential to support the implementation hereof. The secondary recommendations are not mandatory under the EHDS Regulation but are advisable based on insights from the Pilot. Moreover, the recommendations are presented under subheadings reflecting the WHO Toolkit topics and the key Pilot project deliverables, namely infrastructure (primary recommendations), services and applications (primary and secondary recommendations), and standards and interoperability (secondary recommendations). Finally, please note that the recommendations relating to interoperability focused on semantic and technical interoperability, in line with the work of WP8 [21, 22].

## Results

### Recommendations for implementation of HDABs

HDABs will be public entities that shall be designated by each Member State to carry the responsibility of collecting and managing electronic health data intended for secondary use (tasks set out in Articles 55, 57, 58, 59, and 63 [1]). Member States will have the choice of designating one or more HDABs (Article 55 [1]), depending on national circumstances. This may be done by creating new bodies or by appointment of existing public sector bodies. Thus, the tasks of the HDABs may be divided between different HDABs in a Member State. In this case, the Member State must designate one of these HDABs to act as coordinator (coordinator HDAB) (Article 55.1 [1]).

### Infrastructure

The recommendations relating to infrastructure components as enabling factors for the key functions and services to be upheld by HDABs are presented as primary recommendations in Table 1. The obligations of the HDABs relating to properties of the infrastructure are set out mainly in Article 57.1 of the EHDS Regulation [1]. According hereto, HDABs shall carry out the task of facilitating cross-border access to electronic health data for secondary use hosted in other Member States through the HealthData@EU infrastructure. To meet this obligation, the HDABs in each Member State must ensure the connection to their designated National Contact Point (that may potentially be integrated in the coordinator HDAB, according to Article 75.1 [1]) for secondary use of electronic health data (Supplementary Fig. S2) [23]. Each Member State shall designate one single National Contact Point to be an organizational and technical gateway, enabling and responsible for making electronic health data available for secondary use in a cross-border context (Article 75 [1]). It is via this National Contact Point that cross-border connection is established with National Contact Points in other Member States and with the EU Central Platform for HealthData@EU. This distributed network will, by means of eDelivery communication, enable two of the functionalities set out in the EHDS Regulation [1], namely to make available an electronic national dataset catalogue with details about the source and nature of available electronic datasets (data discovery step on the user journey, Fig. 2); and to manage and decide on health data requests and data access applications, including issuing permits for access to electronic health data for secondary use (data permit application step on the user journey, Fig. 2). To facilitate connection of different information systems (e.g. dataset catalogues and data permit management systems) across HDABs, the national side of the IT architecture was designed to be made of three conceptual national components: the Cross-Border Engines, to be integrated in the HDAB, and the National Connector and the Cross-Border Gateway, to be integrated in the National Contact Point (Supplementary Fig. S2). The Cross-Border Engines will serve to translate national standards/interfaces to cross-border standards/interfaces, thereby allowing the HDABs to extend the functionalities of existing information systems to meet those defined by the EHDS Regulation [1].

**Table 1.** Primary recommendations relating to Infrastructure and Services and applications in the implementation of Health Data Access Bodies, based on the HealthData@EU Pilot

**Infrastructure**
**Connecting national IT systems to the HealthData@EU infrastructure and implementing Cross-Border Engines** *Informed by WP5; to adhere to Articles 57.1 and 75 [1]* Member States must connect to the HealthData@EU infrastructure by learning about and deploying the national IT infrastructure components developed by WP5 in the Pilot and maintained and further developed by the European Commission. The HDAB must assess the need for Cross-Border Engines and deploy one for each application, guided by the Architecture Definition from WP5 of the Pilot [23]. To this end, HDABs should ensure that their national applications, such as the IT and information systems of the National Dataset Catalogue and data permit management systems, are developed or adapted to meet the requirements for connection to the HealthData@EU infrastructure.
**Implementing National Connectors and Cross-Border Gateways** *Informed by WP5; to adhere to Articles 57.1(i) and 75 [1]* The National Contact Point, potentially integrated in the coordinator HDAB, must deploy one National Connector and one Cross-Border Gateway. The deployment of both these national components must respect the technical specifications of the HealthData@EU framework and may leverage the open-source components provided by the European Commission [23].
**Services and applications**
**Developing a HealthDCAT-AP compliant National Dataset Catalogue** *Informed by WP6; to adhere to Articles 57.1(j) and 77 [1]* For the HDABs to make available a machine- and human-readable electronic National Dataset Catalogue, it is recommended to either map any current dataset catalogue(s) to or establish a dataset catalogue that is compliant with the specifications introduced by HealthDCAT-AP, as developed by WP6 in the Pilot and made available as open source by the European Commission [24, 25].
**Enhancing HealthDCAT-AP** *Informed by WP6, WP8, and WP9; to adhere to Articles 57.1(j) and 77 [1]* In addition to the HealthDCAT-AP with use of all mandatory properties, HDABs are recommended to assess the need for extending the HealthDCAT-AP to fit specific national requirements and meet the needs relating to different data types in the health domain, e.g. genomic data or imaging data, and level of detail, e.g. metadata at variable level [24, 25]. Knowledge-sharing across HDABs is recommended to save resources and build capacity.
**Adopting common data application forms** *Informed by WP7, WP8, and WP9; to adhere to Articles 57.1(a), 57.1(e), 68, and 69 [1]* To harmonize management of applications for access to electronic health data for secondary use throughout the HealthData@EU infrastructure, HDABs are recommended to leverage the common data application forms, i.e. the data access application form and the data request form, developed by WP7 in the Pilot and made available as open source by the European Commission [26].

The design and provision of these national infrastructure components will be key enablers for the Member States in connecting to the HealthData@EU infrastructure and meet their obligation to facilitate cross-border access to electronic health data for secondary use. Still, the solution developed in the Pilot is not to be considered standalone, and some developmental work and/or procurement will be needed on the national side to connect existing IT systems to the HealthData@EU infrastructure components [19, 23].

### Services and applications

The recommendations relating to services and applications as enabling factors for the key services to be upheld by HDABs are presented as primary and secondary recommendations in Tables 1 and 2. According to the EHDS Regulation, HDABs shall provide a range of services and applications to health data users relating to secondary use of electronic health data across borders [1]. By implementing the national infrastructure components (Supplementary Fig. S2 and Table 1) in the HDAB and National Contact Point, HDABs in each Member State will be able to connect to the distributed infrastructure and the EU Central Platform. This will allow for the exchange of electronic information, e.g. relating to metadata and data permit applications, throughout the HealthData@EU infrastructure and will ultimately provide data users a single point of access to the HealthData@EU.

**Table 2.** Secondary recommendations relating to services and applications and standards and interoperability in the implementation of Health Data Access Bodies, based on the HealthData@EU Pilot

**Services and applications**
**Utilizing eTranslation for National Dataset Catalogues and common data application forms** *Informed by WP7 and WP9; related to Articles 57.1, 68, 69, and 77 [1]* To facilitate cross-border data use while retaining high-quality information, HDABs may consider leveraging the open-source eTranslation solution provided by the European Commission to make available the National Dataset Catalogue also in English and the common data application forms in any of the official EU languages [11, 27].
**Standards and interoperability**
**Strengthening harmonization of validation procedures and data models across Member States** *Informed by WP8; related to Articles 57.1(g), 57.1(h), 57.1(i), and 92 [1]* Member States are recommended to work together in the EHDS Board to strengthen the harmonization of validation procedures and data models across Member States to facilitate the data preparation process and ensure consistent and interoperable datasets [28].
**Providing clear guidelines on semantic and technical interoperability and data quality** *Informed by WP8; related Articles 57.1(g), 57.1(h), 57.1(i), and 82 [1]* Member States are recommended to work together in the EHDS Board to provide and share clear guidelines on semantic and technical interoperability and data quality across Member States. The capacity-building framework laid out in the EHDS Regulation (Article 82) could support the development of guidelines and training modules to promote best practices in relation to data quality [21, 28].
**Leveraging domain expertise and knowledge-sharing** *Informed by WP8 and WP9; related to Articles 57.1(g), 57.1(h), and 57.1.(i) [1]* HDABs are recommended to consider ways to facilitate best use of the different data types included in the EHDS Regulation. This will necessitate expertise within different domains, i.e. experts who have worked ‘close to the data’, as they are key to assisting the semantic interoperability of the data. Knowledge-sharing between HDABs on how to attain this may be beneficial [11].
**Ensuring the ability to link data across domains** *Informed by WP9; related to Article 57 [1]* Member States are recommended to work together in the EHDS Board and collaborate with data holders to ensure that data from different data sources and across data domains are linkable, e.g. the ability to link health data with data on factors impacting on health, such as socio-economic data [11].
**Balancing common data models and data provenance** *Informed by WP8 and WP9; related to Articles 57.1(g), 57.1(h), and 57.1(i) [1]* HDABs can expect some demand on mapping of data to common data models, however they should not provide only mapped data as information can be lost during the mapping process. Also, they are recommended to ensure a level of transparency and information on potential mapping processes, including on data provenance [11, 28].
**Evaluating the use of mock-up or synthetic data for research preparation and validation** *Informed by WP8 and WP9; related to Article 57 [1]* HDABs are encouraged to evaluate the potentials for providing mock-up or synthetic data to support research preparation and validation. Future common properties of HealthDCAT-AP, such as subsets of data, e.g. synthetic, may be developed in collaborative efforts [28].

The EHDS Regulation requires HDABs to provide a national dataset catalogue, i.e. metadata about available datasets, detailing their source, scope, main characteristics, data nature, and access conditions (Articles 57.1 and 77 [1]). To facilitate data discovery, a standardized descriptive metadata model, the HealthDCAT application profile (HealthDCAT-AP), fit for the purpose of the EHDS Regulation was developed and implemented in the Pilot [24, 25]. The national metadata catalogues of the HDABs must comply with the specifications introduced by HealthDCAT-AP. HDABs will have a supervisory and facilitating role in relation to data holders who provide the metadata for the catalogue. For the metadata catalogue to cover the needs of data users, there is a need for combining the national dataset catalogue with data quality and utility labels [11, 19]. The properties of HealthDCAT-AP provide the necessary metrics for HDABs to generate data quality labels from metadata.

In the Pilot, an EHDS and General Data Protection Regulation (GDPR) compliant common data access application form and a data request were developed with the aim of harmonizing the structure and content of such forms across Member States [26]. The common data access application form was designed to be used for applying for anonymous or pseudonymized personal-level data (Article 67 [1]), while the data request was designed to be used for requesting anonymous aggregated data (Article 69 [1]). These forms were designed to cover all the relevant pieces of information for the HDABs to assess whether to grant data applicants access to electronic health data through the HealthData@EU (Article 68 [1]). The implementation of the health data request is expected to simplify the data access processes and alleviate approval bottlenecks that delay research. As such, when HDABs provide answers to health data requests, the aggregated data can allow the applicant to conduct initial analyses to refine their study datasets before potentially applying for a data permit. The data access application form has been integrated in the data access request portal prototype at the EU Central Platform and connected to the EU Dataset Catalogue. The objective is to provide for a single entry point for data applicants to fill in data access application forms that can then be dispatched to the national data permit management system of the relevant HDABs via the HealthData@EU infrastructure (Supplementary Fig. S2). Moreover, the IT solutions provided by the Pilot enable status updates of an application [23]. The importance of continuous evaluation of the functionality and fitness for the purpose of the common data access application form as a collaborative effort between HDABs, data holders, data applicants, and ethical and scientific committees was underlined to ensure the feedback required for adjusting and further developing the common data access application form as the HealthData@EU is being implemented.

### Standards and interoperability

The recommendations relating to standards and interoperability (semantic and technical) as enabling factors for the key functions and services to be upheld by HDABs are presented as secondary recommendations in Table 2. The obligations of the HDABs relating hereto are set out mainly in Article 57.1 of the EHDS Regulation [1]. The Pilot has underlined that harmonization of validation procedures and data models across Member States is essential for consistent implementation of interoperability standards, ensuring semantic interoperability and ultimately high-quality studies [21, 28]. Such harmonization will support not only the interoperability in single multinational studies but will also ease data preparation and semantic and technical interoperability in relation to public health surveillance, relying on consistent national reports to produce accurate, EU-wide insights [11]. Providing data quality checkpoints at multiple stages of data aggregation can also help data users verify the accuracy of data across countries. Additionally, consistent documentation of data provenance is considered critical for reusability, trust, and transparency in health data research [28]. However, while establishing a chain of provenance for data is essential, it remains challenging as it involves detailed documentation of the origin and handling of data, and thus, requires significant and continuous efforts and standardization throughout the health data ecosystem.

## Discussion

This article presents recommendations based on the work of WP4 in the Pilot to support the implementation of HDABs within the HealthData@EU infrastructure. Being a horizontal work package, these recommendations aimed to provide an overall synthesis of the Pilot project’s achievements to address key enablers for HDAB implementation and ability to facilitate secondary use of electronic health data across Member States.

The Pilot demonstrated both many of the current barriers for secondary use of electronic health data and the potential of carefully implementing the EHDS Regulation to help reduce these obstacles. Thus, while great efforts will be required beyond the Pilot, including in the national adaptation of the EHDS Regulation, the implementation of the HealthData@EU infrastructure with HDABs as key entities holds the potential of providing substantial benefits for healthcare research, public health monitoring, and policymaking across Europe [5, 8]. The recommendations developed from the Pilot drew on the experiences of technical work packages and Pilot use cases, aiming to streamline processes for health data discovery and access, improve semantic and technical interoperability, and establish a framework for cross-border data sharing. The Pilot highlighted specific areas of improvement that may greatly benefit data access, notably data discovery and the data permit application process. This process, often experienced by data users as highly challenging and time-intensive in cross-border studies [11], is foreseen to benefit from harmonization, such as standardized application forms and a centralized application system. These changes may enhance the user journey by reducing administrative burdens and simplifying data access for data users across Member States.

The implementation, maintenance, and long-term success of HDABs rely on a range of dependencies. Successful establishment and operation will demand a multidisciplinary workforce with specialized skills in data management, IT infrastructure, and legal compliance to deploy the Pilot project’s outputs, namely the IT infrastructure components, the common data access application forms, and a HealthDCAT-AP compliant national metadata catalogue [10, 19]. Article 55.2 of the EHDS Regulation mandates that Member States provide HDABs with adequate human and financial resources and the necessary expertice [1]. However, hiring and retaining critical workforce competencies will expectedly be a central challenge for HDABs to overcome given that technical expertise in infrastructure development, metadata management, data security, and semantic and technical interoperability together with legal expertise are in high demand in a competitive labour market and often, additional training will be required to meet specific requirements. Addressing this will require Member States to explore collaborative training programs and consider partnerships, e.g. with universities, for workforce development to bridge gaps in expertise. Furthermore, establishing well-defined governance structures and clear organizational mandates are essential to effectively integrate HDABs within national health systems and ensure transparency. Interdisciplinary skills, especially in project management, will be crucial for HDABs to coordinate with other institutions and European entities, facilitating consistent application of the EHDS Regulation across borders. The HDAB must also ensure competences with a high degree of knowledge about different data types and be in continuous dialogue with data holders to ensure HDABs are well-prepared to manage diverse health data types and comply with legal requirements. Member States are encouraged to leverage the EHDS Regulations’ capacity-building provisions (Article 82 [1]), which promote best practices and shared expertise.

The Pilot project’s context and timing, and the methodology behind these recommendations presented certain limitations. Consultations occurred while many Member States were still in preliminary HDAB planning phases and negotiations on the EHDS Regulation were ongoing, introducing political sensitivity and restricting the ability to fully assess long-term organizational and workforce impacts. Following, many questions remained unanswered in relation to the actual implementation of the EHDS, both regarding primary and secondary use, and were outside the scope of the Pilot. Moreover, the Pilot primarily drew on use cases relying on pre-existing infrastructures. These factors imply that additional, unforeseen challenges may emerge as HDABs begin large-scale operations, highlighting the need for ongoing refinement and adaptability in HDAB infrastructure.

The recommendations provided here were intentionally high-level, acknowledging the diversity in healthcare digital maturity and data governance models across Member States. Flexibility in implementation will be necessary to accommodate each country’s unique needs and infrastructure. Following, the recommendations presented in this article and additional recommendations relating to the topics of Organization and governance and Workforce included in the WP4 Recommendations Report [10], may serve as a framework that Member States and HDABs can customize, with collaborative support from successive actions and activities concerning the EHDS, the HDABs-CoP as a common collaborative platform between national HDABs [14], and the European Commission, especially in the future EHDS Board (Article 92 [1]).

## Conclusion

The implementation of HDABs within the HealthData@EU infrastructure and the EHDS framework represents a transformative step towards a more accessible health data ecosystem across Europe. Key aspects regarding the roles of HDABs have been clearly set out by the EHDS Regulation, and the Pilot provided several solutions to be deployed by HDABs in all Member States. Also, the Pilot shed light upon aspects of the HDAB implementation that remain ambiguous, to be addressed among Member States in subsequent projects and communities, or to be settled at the national level. Thus, careful and successful implementation greatly depends on intensive collaboration at both national level in each Member State and at EU level.

## Supplementary Material

ckaf033_Supplementary_Data

## Data Availability

The data underlying this article are available in the article, in its references, and in its online supplementary material.

## Supplementary data

Conflict of interest: C.F.C. and L.S.S. are employees at the Department of Clinical Epidemiology, Department of Clinical Medicine, Aarhus University and Aarhus University Hospital, Denmark. The Department of Clinical Epidemiology is involved in studies with funding from various companies as research grants to and administered by Aarhus University. None of these studies are related to the current study. C.F.C. and L.S.S. declare no personal conflicts of interest.

## Disclaimer

Key points

- Health Data Access Bodies (HDABs) are essential public entities designated by EU Member States to manage and facilitate secondary use of electronic health data across borders, supporting the goals of the European Health Data Space (EHDS) within the HealthData@EU infrastructure.
- Recommendations for HDAB implementation focus on establishing a robust infrastructure, interoperable services, and standardized procedures to ensure efficient cross-border data access and compliance with EU regulations.
- A standardized national dataset catalogue, based on the HealthDCAT-AP metadata model, is advised to enhance data discovery across Member States.
- Common data access application forms will simplify data permit application and data request procedures, facilitating timely access to data for research, policy, and public health applications.
- The implementation of HDABs within the HealthData@EU infrastructure and the EHDS framework represents a transformative step towards a more accessible health data ecosystem across Europe.
