# Supplementary material for: Shaping the future EHDS: recommendations for implementation of Health Data Access Bodies in the HealthData@EU infrastructure for secondary use of electronic health data
Source: Eur J Public Health. 2025 Sep 10;35(Suppl 3):iii32–8. doi: 10.1093/eurpub/ckaf033 (PMC12420904; doi:10.1093/eurpub/ckaf033)
Supplement: ckaf033_Supplementary_Data [file ckaf033_supplementary_data.pdf]

Supplementary materials

Shaping the future EHDS: Recommendations for implementation of Health Data Access Bodies in the HealthData@EU infrastructure for secondary use of electronic health data

Lise S. Svingel, Caroline E. Jensen, Gitte F. Kjeldsen, Maria H. Pedersen, Dipak Kalra, Christian F. Christiansen, Katrine H. Vad

Contents

Supplementary tables 2

Table S1. *List of WP4 activities in the HealthData@EU Pilot project* 2

Supplementary figures 4

Figure S1. *Overview of data collection* 4

Figure S2. *Reference implementation for the HealthData@EU infrastructure (modified from WP5 of the HealthData@EU Pilot and reprinted with permission from the HealthData@EU Pilot)* 5

Supplementary tables

Table S1. *List of WP4 activities in the HealthData@EU Pilot project*

**Activities**			**Participants, contributors, or respondents**	**Perspective**
**Type**	**Target group**	**Place and time**	**Affiliation(s)**	
**Meeting / consultation**	HealthData@EU Pilot consortium	Online, monthly		HDAB, Data user
	HealthData@EU Pilot project coordinators (WP1)	Online, monthly	Health Data Hub (public)	Data user, HDAB
	HealthData@EU Pilot use case managers (WP9) and use case leaders	Online, monthly		Data user
	European Commission, DG Santé	Online, ad hoc	European Commission, DG Santé (public)	
	External Advisory Board	Online, 16 January 2024	France Digitale (private)	
			Heva, Docaposte (private)	
			Sundhed.dk (public)	
			The European Institute for Innovation through Health Data (private-public)	
			Promptly Health (private)	
			CETIC (private)	
		Online, 24 May 2024	France Digitale (private)	
			The European Institute for Innovation through Health Data (private-public)	
			Promptly Health (private)	
			Digitale Europe	
**Workshop**	HealthData@EU Pilot consortium	Paris, 20 October 2023		HDAB, Data user

		Paris, 18 October 2024		HDAB, Data user
	HDAB Community of Practice[8]	Brussels, 26 January 2024		HDAB
**Questionnaire**	HealthData@EU Pilot use case participant and leaders	Online		Data user, data holder
	HDAB Community of Practice[8]	Online		HDAB
**Interview**	National authorities from Member States participating in the Community of Practice[8]	Online, 16 May 2024	Croatia (public)	HDAB
		In person, May 2024	Denmark (public)	HDAB
		Online, 15 May 2024	Ireland (public)	HDAB
	HealthData@EU Pilot technical work package leaders (WPs 5-8)	Online, May-July 2024	WP5	HDAB, Data user
			WP6	HDAB, Data user
			WP7	HDAB, Data user
			WP8	HDAB, Data user

Supplementary figures

Figure S1. *Overview of data collection*

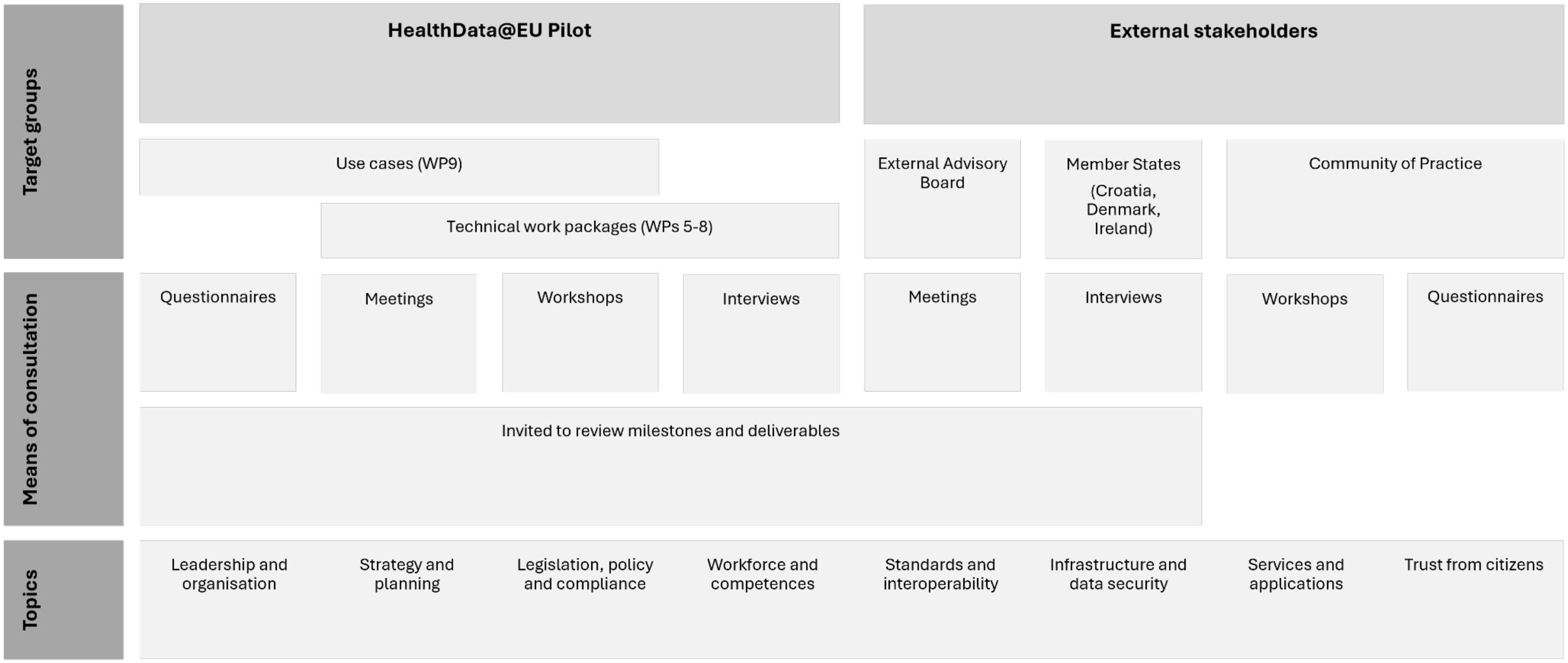

Figure S2. *Reference implementation for the HealthData@EU infrastructure (modified from WP5 of the HealthData@EU Pilot and reprinted with permission from the HealthData@EU Pilot)*

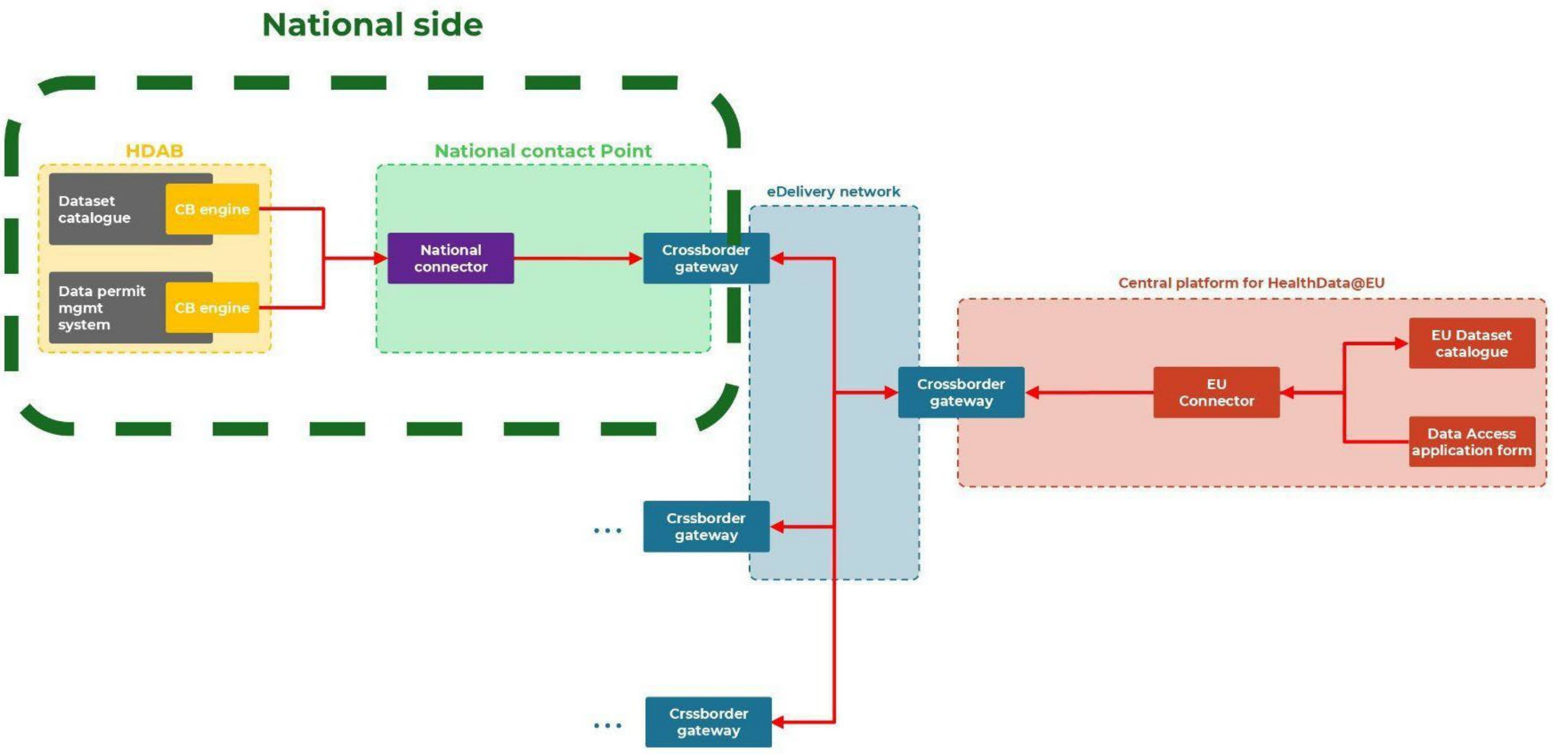

Abbreviations: CB engine: Cross-Border Engine; HDAB: Health Data Access Body
